# 

**Published:** 1921-03-12

**Authors:** 


### THE HOSPITAL

The Modern Newspaper of Administrative Medicine and Institutional Life.

Telegraphic Address: OSPEDALE, RAND, LONDON. Telephone Number: 2734 GERRARD.

No. 1814. Vol. LXIX. | Saturday, March 12th, 1921. | PRICE TWOPENCE.

